# Living Creatively, In and Through Institutions

**DOI:** 10.5964/ejop.v12i1.1133

**Published:** 2016-02-29

**Authors:** Tania Zittoun

**Affiliations:** a University of Neuchâtel, Neuchâtel, Switzerland

## From Basic Processes to Existentially Significant Questions

Psychologists, as many social scientists, try to describe and understand what is to live as humans in society. If many were interested and fascinated by distant social groups, or studied them for the reflexive distance their understanding allowed, there is still great need to understand our current social, cultural and political environment. This is a new task for many psychologists, because their discipline has dominantly preferred to examine “small” and clearly cut problems over the last fifty years: task resolution, attachment, attitude or reasoning. Attempts to address serious and complex problems, such as Goffman’s work on total institutions (Goffman, 1961) or Jodelet’s study of madness in a French village (Jodelet, 1989) are considered as marginal if not off-discipline, and other attempts such as Milgram’s to understand the conditions of Nazism through submission to authority (Milgram, 2009) are currently very critically reassessed (Brannigan, Nicholson, & Cherry, 2015; Mastroianni, 2015).

More recently, and coming from different critical horizons – social psychology, political psychology, critical psychology, cultural psychology, dialogical approaches in psychology – many dare to address “big” questions. These always demand an understanding of the person-in-context, in relationship to others and their environment, and address complex psychological experiences (Molenaar, 2004; Valsiner, Marsico, Chaudhary, Sato, & Dazzani, 2016). Among these, authors have examined the experience of trust in one’s social environment (Marková & Gillespie, 2008, 2011), the experience of growing up in conditions of great poverty (Bastos, Rabinovich, & Valsiner, 2009), of living in conditions of social and political transformation (Daiute, 2010; Magioglou, 2014; Wagoner, Jensen, & Oldmeadow, 2012), of migration and losing one’s home (Bhatia, 2011; Kadianaki, 2009; Märtsin & Mahmoud, 2012), or more simply – but not less complex – the experience of daily life in a complex society (Dreier, 2007; Schraube & Højholt, 2016).

Transversal to many of these studies, an important challenge reappears: how can we fully understand how people deal with institutions, and how institutions guide, channel, enable or at times constrain people’s trajectories? And conversely, how to account for the basic or minimal conditions that allow people to find their own ways, or confer sense to their experience within institutions? It is these two questions, often reduced to the structure-agency or the determinism-freedom problems, that I wish to address. Rather than a full theorization, I propose a sketch that may have a programmatic function.

## Theorizing Institutions

The question of how to account for the “context” or the “environment” in which people live and their constraining role is not new in psychology, by any means (for reviews see Grossen, 2001; Marková, 2015). These attempts have usually drawn on abstract theories and models to then sketch complex systemic models, for instance to address the development conditions of the child (Bronfenbrenner, 1979; Van Geert, 2003) or the working conditions of the adult (Engeström, Engeström, & Kärkkäinen, 1995). Combined with this, other theories have tried to qualify the “effect” of the sociocultural environment on influencing people (Ratner, 2014), framing lives (Goffman, 1974; Grossen & Perret-Clermont, 1992), channeling or guiding conduct (Toomela, 2003; Valsiner, 2008), or some complex combination of giving shape to people’s motives, favoring certain means to get to some end, and shaping the meaning of actions (Abramson, 2012).

But how to specifically theorize the role of institutions in psychology? Interestingly, neither the *International encyclopedia of the social and behavioral sciences* (Smelser & Baltes, 2001), nor the more recent *Encyclopedia of critical psychology* (Teo, 2014) (nor any of the reference works consulted in various fields of psychology) define the term ‘institution’ (but some individual studies have, see for instance Marková, in press) Sociology had, in contrast, defined itself as the study of institutions, understood as “any system of beliefs, or collective modes of actions” (after Durkheim, 2002, p. 15, my translation). And so these can be defined as such:

An institution is the fixing of stereotyped social interactions in the form of rules. In most cases these rules are made explicit and there are sanctioning mechanisms behind them. Yet sometimes these characteristics are absent, for example when people adhere to such rules simply because they feel urged to act in this way. An institution does not need to be a large organization. The largest institutions known are states or multinational organizations like the United Nations, yet there are also much smaller institutions, as for example marriage or monthly meetings of a group at a certain pub. Therefore, institutionalization is not a matter of size. (Henning, 2007)

It is striking that this sociological definition is imbued with psychological explanation: people “simply feel urged to act in this way” (above). How can we reread such proposition? We will first comment on the sociocultural nature of institutions, before turning to people’s experiences.

From a sociocultural psychological perspective, which emphasized the semiotic, material and temporal nature of the sociocultural environment (Valsiner, 2014), we could say that institutions are temporarily solidified meanings and patterns of interactions, which are usually crystallized in different forms: materiality (as the walls of a school), semiotic constructions (as written regulation or textbooks) or ideal or communicational constructions, such as social representations. As solidified patterns, these usually change more slowly than other sociocultural realities – unless there is of course a sudden revolution, which reaccelerates the change (Zittoun & Gillespie, 2015), or important changes in the general conditions of life (e.g., a financial crises, see for instance Ratner, 2014). From that perspective, different institutions do not differ in size, but in the nature of their composition, and therefore, resistance and inertia. In effect, it might be useful to distinguish institutions that are strongly bounded to a material setting, such as a school or a ministry, or more fluid institutions, such a football matches, that are highly regulated yet can take places in different places and dissolve again (Zittoun et al., 2013, pp. 144-145). Anyhow, what counts here is that institutions are usually “given” to people by the social environment. Because of their material and symbolic composition and their particular temporality, they tend to be transmitted across generations. These are therefore often perceived by people that meet them as what is “out there” and that escapes to some extend to individual action. Of course, constructionist approaches have shown us how much everything is constructed (Berger & Luckmann, 1966); more practically, all institutions are human made, and people usually often have more power than they think to participate to their evolution – whether explicitly in democratic societies, or through active or passive resistance in other situations.

Institutions create only the setting for people’s action and meaning making. Institutions are given to many people, yet people do not experience institutions similarly: even the most totalitarian institutions do not turn every person into a copy of their neighbor. This is where it is useful to distinguish the socially given from the psychologically experienced, as indicated by phenomenological psychosociology (Lewin, 1936, 2000; Schuetz, 1944, 1951). Drawing on this approach, we have thus distinguished the socially and materially given – the settings – from how people experience specific situations. We have used the term “spheres of experience” to designate this more subjective experience of being engaged in a recurrent pattern of activity, feeling, intentions, meanings, etc. This includes for example the sphere of experience of having a family dinner (which can take place in different physical spaces and around different dishes, yet feels quite “the same” for a given person over time as it has a certain emotional quality, touches identity aspects, demands the mastery of informal rules, or the sphere of experience of reading one’s students’ emails – which can take place in the same room or even at the same table where one does many other activities, yet which demand specific skills and orientations (Zittoun & Gillespie, 2015, 2016). This important distinction – between what is given and what is experienced – is central both to identify how the first one enables and constrains the second, and how people can still maintain freedom within constraining conditions.

## The Work of Institutions

Now that we have defined institutions, we have to identify the specific dynamics by which institutions, as sociocultural constructs, participate to the definition of people’s lives. Like any other form of cultural constructs, institutions can act upon people from within, and from without. From within, in effect, they provide values, discourses and actions that have shaped the person’s conduct and that she or he has internalized and that can now guide her understanding of the world and her externalization. They also guide from without, because institutions create settings for interactions and the modalities of recognition of the person. It is thus possible to speak about a double orchestration of human conduct (Baucal & Zittoun, 2013). If we give a closer look at institutions, however, we need to identify at least a triple guidance.

First, as they have been shaped through time, participate to a certain society, present people redundantly with certain discourses and ways of doing, they participate to the socialization of persons, the activities they develop, and therefore the semiotic means they have internalized. Internalization itself of course can be more or less deep (Valsiner, 1998). Some values or discourses can be simply repeated by a person and forgotten. Others can be consciously or deliberately called upon if necessary. Still others can become deeply internalized and recombined and generalized through layers of a person’s experience. These become so to say transparent to the person – as can, for instance, be the respect to authority, or certain non-conscious ways to experience gender (Ratner, 2014). Others can even be internalized and repressed by other layers, and thus appear indirectly, for instance through dreams (Beradt, 2004; Zittoun, 2011) or other manifestation of unconscious material.

Second, institutions create time, material places and spaces, interactions and activities. A person’s action is thus guided in and by daily interactions. For instance, people in retirement home have to come to dinner at certain hours, move in specific spaces, obey to security instructions, or interact with health carers at certain imposed moments (Salamin, 2015). Concretely, thus, daily conduct is punctuated by strongly guided encounters. In each interaction, certain conduct, aspects of identity or attempt to confer meaning to the situation are encouraged, guided, validated by others, or at the contrary, not recognized, or forbidden. This can (but must not) change future conducts, or shape the internalization that will follow. Hence, for instance, teachers can, during their interactions with students, make them feel that their way to invest a book or a movie is inadequate. Students can feel that lack of recognition and disinvest learning (Zittoun, 2014b). Yet there also, people have usually unguided periods of time: moments to rest, or without surveillance, or breaks; little escapes watching out of the window, or bigger, leaving the walls of an institution; and people can learn despite repeated teachers’ rebuff (Mehmeti, 2013).

Third, as a whole, representatives of institutions can also produce and diffuse discourses and other crystallized traces based on these interactions with specific persons – they produce institutional artefacts. These artefacts are most likely to be used by further representatives of institutions as resources when meeting the same person, further in his or her lifecourse. Hence, schools produce grades which will be used by college admission boards, workplaces produce recommendation letters that will be used by future employers, doctors produce evaluations that will be used by insurance agents, or, as it is the case in certain regions of Switzerland, police officers produce reports that can be used by civil officers in charge of deciding if the resident can receive the local nationality (Zittoun, 2015). It is partly because the work of so many agents or gatekeepers of institutions rely on previous colleagues’ work that social reproduction (for example in education) can so blindly be conducted, or that administrative handicapping of life can be produced (Mehan, Hertweck, & Meihls, 1986). Finally, it is also because museums buy the paintings of an artist or schools put a bust of their teachers in the hall that some people become socially acknowledged and thus part of the “institution”. Yet here also, there are always teachers that decide not to consult the students’ past records (Zittoun, 2004), doctors that reassess the health of their new patients, or, at times, migrants that can have their application reexamined (Di Donato, 2014). There are also authors that refuse Nobel prices (such as Sartre) and thus escape becoming representative of institutions.

These three aspects of institutional guidance – semiotic guidance, situated interactions and produced artefacts – can, of course, be more or less adjusted and consonant, or dissonant. Institutions may promote values which are not put into daily interactions, or interactions might lead to expectations which are not followed up by the cultural artefact they produce. People usually feel these discrepancies, and experiences them as lack of justice, or absurdities – and these might be the origins of questions and the trigger for imagination and action, as we will see.

It is important to note that, although the guidance of institutions is pervasive, it is not only negative. Institutions are usually the warrant of some aspects of a cultural system and thus participate to its maintenance, transmission and renewal. Schools give people means to learn to read and think, administration allows for social security, and hospitals still help to preserve personal health. Whether these institutions treat people unequally, take advantage of their power, is a problem that needs to be addressed; yet the total lack of institutions is also the end of peaceful coexistence in a complex world. As much as the ecosystem is the condition of our biological existence as humans while being at times hostile, institutions are the basic conditions of our cultural lives, before being constraining or limiting freedom.

## People’s Creativity in Living

Institutions have a triple way to channel and guide people’s conduct and sense making, from within and from without. Yet, as already suggested, people are most of the time not reduced to what institutions impinge on them, even when they do not actively resists or oppose an institutional order. Students develop (sometimes) autonomous and critical thinking, old people in retirement homes define their “corner” in an unused corridor and feel at home, employees can enjoy their work as well as join workers unions to make propositions to their authorities, and prisoners escape their prisons in their daydreams, or materially. Either way, people do not simply comply.

Scholars have proposed different notions to designate people’s capacity to stand as persons in front of, or within institutions; they have used the terms self-determination, autonomy, agency, subjectivity, and so forth. As these terms have all complex underpinnings which I will not discuss here, I would like to propose another one – that designates people’s creativity. For this, I will draw on Winnicott’s propositions:

It is creative apperception more than anything else that makes the individual feel that life is worth living. Contrasted with this is one of compliance, the world and its details being recognized but only as something to be fitted in with or demanding adaptation (Winnicott, 2001, p. 65).

As a psychoanalyst and pediatrician, Winnicott was interested in how people, sometimes in very difficult situations, could maintain their strive for life; he called creativity a specific meeting of the self and the world, by which one could have the experience of having “created” the given. Grounded in early interactions, that experience expands in adulthood in the repeated experience we may have of seeing the world as-if it were new – seeing the tree at of one’s window as if it was for the first time. This experience is thus that of being proactive in one’s relation to the world, and not simply reacting – elsewhere, Winnicott opposes it to the response to a stimulus (Winnicott, 2004). This is partly because this creative response engages the person, with all her past experiences, memories and embodied emotions. I do believe that Winnicott thus captured something very important about people’s engagement in their spheres of experience. My proposition is that there is something of this creativity that allows people not to be over-burdened by the triple guidance of institutions.

Basic creativity allows people to experience the institutions that they come to interact with or in not only as constraining, but also enabling; not only as imposing, but also as providing them with means to express themselves, explore their world, or experience their capacities and skills. More specifically, such creativity may allow people to live a meaningful life within or facing institutions in four related ways. First, it is the basis of a playful relation to the given, and thus allows defining an acceptable sphere of experience in a given setting: a possible way of understanding the classroom, a comfortable promenade in the retirement home, or a trustable colleague in the workplace. Second, it is indeed the basis of imagination, thus allowing the exploration of distal experiences – imagining alternatives, distant, past or future experiences (Zittoun & Gillespie, 2016). As such, imagination and creativity allow simply writing a good essay, thinking about the future life of one’s grandchildren, or design a new piece of machinery; it also allows escaping institutional constraints through daydream, or parallel or underground activities. This might, third, lead to life-creativity (Zittoun & de Saint-Laurent, 2015) – decide to quit an institution and reorient one’s life. It may finally even allow, fourth, proposing alternatives to the institutional guidance: oppose a grade, object an administrative decision, organizing a demonstration, or with others, propose an innovation or develop a new political imagination (Glăveanu & de Saint-Laurent, 2015).

It is important to note that such understanding of existential creativity goes beyond a naïve acceptance of one’s life conditions, and should not be reduced to the “symbolic violence” of accepting and internalizing otherwise oppressive powers (against Ratner, 2014). In effect, people do not come naked in front of any institutions: they experience it as one in a long series of spheres of experience they will or they have met. People develop through time, and through many social settings; only in extreme cases do people live in total institutions. This implies that, over time, people internalize different values, meanings and discourses from different spheres of experience. It is with these layers of more or less integrated experiences that people interact with institutions. Their past trajectories lead them to experience tensions and contradictions within what is offered to them, or between what is proposed and their past and imagined experiences, or can give them resources to deal with new demands (Gillespie, Cornish, Aveling, & Zittoun, 2008; Gillespie & Zittoun, 2015; Zittoun & Gillespie, 2015). It is the layered nature of human experience that allows inner dialogue (Marková, 2006; Zittoun, 2014a), and with it, such existential creativity. This is why being creative in an institution is not submitting to it; it is the way to meet it as unique person, facing some of the cultural construction of others – which need of course sometimes to be opposed.

## A Programmatic Stance

The reading proposed here suggests that given the fundamental role of institutions in our lives in societies, it might well be time for psychologists to fully understand how they function, to further explore their triple modality of guidance, as well as how people interact with them, and how they develop their spheres of experiences within and across them. Consequently, it is also very important for psychologists to understand at what conditions an existential creativity can be preserved or restored in every person.

When basic conditions of safety, emotional security, recognition, dialogue, are threatened, great strains are put on people and their experience of creative life. When people choose, or feel forced, to comply to any institutional order – even well-intentioned ones – then societies are in trouble. In effect, without individual creativities, people cannot participate to the renewal, questioning and necessary correction of institutions. Hence, if people loose individual creativity, societies are at risk of losing much more. Conversely, when people feel too threatened, or in risk of alienation, they might also reject all societal institutions as well. If their permanent renewal is necessary, it might be preferable for all if this remains a dialogical process (Marková, in press).

Learning from small and large institutions, present and past, we may have better tools to understand what is at stake for people’s trajectories in the general shivering of our institutions. This might be one of the tasks for psychologists in Europe today.
